# Disentangling host identity and storage time effects on gut microbiota composition in captive migratory birds using absolute and relative quantification

**DOI:** 10.1093/cz/zoaf066

**Published:** 2025-10-03

**Authors:** Cuiping Liu, Zhiyong Wu, Dongtao Li, Jiaxiao He, Guoliang Li, Fuwen Wei

**Affiliations:** Jiangxi Provincial Key Laboratory of Conservation Biology, College of Forestry, Jiangxi Agricultural University, No. 1101, Zhimin Road, Qingshanhu District, Nanchang 330045, China; Nanchang Zoo, No. 1288, Taohua South Road, Xihu District, Nanchang 330025, China; Nanchang Zoo, No. 1288, Taohua South Road, Xihu District, Nanchang 330025, China; Nanchang Zoo, No. 1288, Taohua South Road, Xihu District, Nanchang 330025, China; Jiangxi Provincial Key Laboratory of Conservation Biology, College of Forestry, Jiangxi Agricultural University, No. 1101, Zhimin Road, Qingshanhu District, Nanchang 330045, China; Jiangxi Provincial Key Laboratory of Conservation Biology, College of Forestry, Jiangxi Agricultural University, No. 1101, Zhimin Road, Qingshanhu District, Nanchang 330045, China; CAS Key Laboratory of Animal Ecology and Conservation Biology, Institute of Zoology, Chinese Academy of Sciences, No. 1, Beichen West Road, Chaoyang District, Beijing 100101, China

**Keywords:** 16S rRNA sequencing, absolute quantitation, captive migratory birds, generalist species, rare taxa, storage time

## Abstract

Understanding how gut microbiota support migratory birds is essential; yet, fecal sample freshness is often a challenge, particularly for rare species that cannot be captured directly. Here, we collected fecal samples from multiple captive migratory bird species at Nanchang Zoo and grouped them by post-defecation time (0, 1, 2, and 4 h). Using both relative and absolute quantification, we assessed the effects of host identity, short-term storage, and their interaction on gut microbiota composition. Species identity and quantification method significantly shaped microbiota profiles. Absolute quantification revealed Firmicutes (763,405.73 copies/μL) and Proteobacteria (340,231.03 copies/μL) as dominant in Grey-crowned Cranes, whereas relative quantification indicated Firmicutes (96.74%) predominated in Swan Geese and Proteobacteria (30.30%) in Black-necked Cranes. Red-crowned Cranes showed higher species richness than Black Swans and Swan Geese, with a significantly greater Shannon index than the latter. PCoA demonstrated clear interspecific differences, especially between crane and waterfowl lineages. Storage time had no significant effects on alpha and beta diversity across 6 species, except for reduced richness in Swan Geese at 2 and 4 h. While overall community structure was stable, a few conditionally rare taxa displayed time-sensitive shifts shortly after defecation. Our findings highlight that both host identity and quantification approach are critical determinants of avian gut microbiota profiles and emphasize that fecal sample freshness mainly affects rare taxa. This study provides methodological insights for optimizing fecal sampling protocols in field-based microbiome research on migratory birds.

## Graphical Abstract

**Graphical Abstract:**
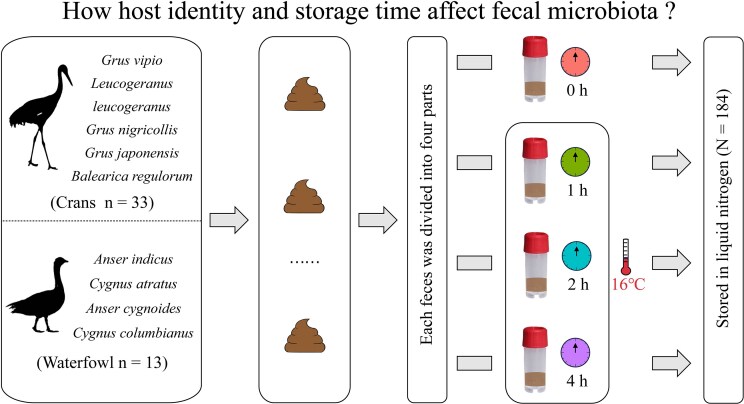

Birds harbor complex gut microbial communities that have coexisted and evolved with hosts over time and play critical roles in host nutrient absorption (Borda-Molina et al. 2018), immune system function (Maki et al. 2019), and metabolic capacity (Apajalahti 2016). A large number of studies have shown that both intrinsic (e.g., genetics, physiology, sex) and extrinsic factors (e.g., habitat, behavior, diet) can sharply affect the composition of gut microbiota (Hird et al. 2015; Corl et al. 2020; Davidson et al. 2020; Lee et al. 2020; Góngora et al. 2021). The relationship between gut microbiota and the host is highly complex, and disruptions or dysbiosis within the microbial community can significantly impair host fitness and offspring quality (Kohl et al. 2018; Somers et al. 2023), potentially leading to death or even population decline (Ambrosini et al. 2019).

Bird migration is one of the most captivating natural phenomena, with approximately one-fifth of the world's nearly 10,000 bird species migrating annually between breeding and wintering grounds (Kirby et al. 2008). During migration, the avian gut microbiota shifts not only with diets and reproductive status but also with key external environmental factors including habitat quality, seasonal changes, pathogen exposure, and anthropogenic pressures, to meet the physiological demands of long-distance travel such as energy regulation and immune defense (Risely et al. 2018, 2017; Skeen et al. 2021; Thie et al. 2022; Trevelline et al. 2023). Studying the avian gut microbiota is often accomplished through fecal samples, a convenient and non-invasive alternative. The gut microbiota of wild birds remains comparatively understudied than that of humans and laboratory animals, partly because it is difficult to obtain fresh, uncontaminated fecal samples in the field. Recent studies have focused on small, non-protected birds whose feces can be collected immediately, whereas feces from larger or rare species are typically collected hours after deposition near resting or foraging sites (e.g., Turjeman et al. 2020; Skeen et al. 2021; Wang et al. 2024). During this period, exposure to environmental factors such as UV radiation and oxygen may alter microbial composition, leading to fecal samples that do not accurately reflect the host's gut microbiota. Therefore, it is essential to determine the optimal time for collecting bird feces after defecation to ensure reliable gut microbiota profiling.

In microbiome research, 2 commonly used methods are relative and absolute quantification, which provide measures of relative and absolute abundance, respectively. While relative abundance data can reveal changes in community structure, they cannot distinguish whether observed differences are due to an increase in a particular taxon, a decrease in others, or a combination of both. Feng et al. (2023) compared chicken gut microbiota using absolute and relative abundance approaches and observed clear discrepancies: the absolute abundance of Firmicutes remained stable across intestinal segments, while its relative abundance in the duodenum and cecum varied significantly due to changes in other phyla, suggesting that the absence of absolute abundance may lead to misinterpretation of community dynamics. Recent studies have shown that absolute abundance measurements provide a more accurate understanding of the true drivers of microbiota changes and avoid the biases associated with relying solely on relative abundance data (Vandeputte et al. 2017; Rao et al. 2021; Bodawatta et al. 2022; Maghini et al. 2024). Compared with traditional absolute quantification methods, spike-ins have become an important tool in microbiome studies due to their broad applicability, high sensitivity, low cost, and ability to improve quantification accuracy (Sender and Milo 2016; Wang et al. 2021; Zaramela et al. 2022).

Understanding how gut microbiota of birds relates to environmental change is crucial for future birds' conservation and management. Due to differences in diet and lifestyle, their gut microbiomes vary significantly. For example, the gut microbiota of the common crane (*Grus grus*) comprises approximately 226 OTUs (Li et al. 2024), whereas that of the whooper swan (*Cygnus cygnus*) harbors an average of 1, 317 OTUs (Fu et al. 2023). Bacterial communities with varying levels of diversity may exhibit distinct patterns of change in response to storage time under specific temperature conditions. Most studies primarily investigate how different storage conditions, such as storage time, temperature and preservation media, affect the stability of microbial communities in a single species, with limited research on multiple species simultaneously (Song et al. 2016; Han et al. 2018; Chen et al. 2019; Maghini et al. 2024). To investigate the role of gut microbiota in different bird species adapting to environmental change, it is important to assess the accuracy of gut microbiota data obtained from field-collected fecal samples from a multi-species perspective.

In natural ecosystems, microbial species are unevenly distributed—some occur at high abundances, while others persist in low or even trace amounts. Rare and abundant taxa may respond differently to environmental disturbances, largely due to variations in their dispersal patterns (Liu et al. 2015) and community assembly processes (Jiao et al. 2019). Additionally, based on ecological niche breadth, microbial species can be classified into generalists or specialists. Generalist species have a broad ecological niche and can adapt to a variety of environments and resources, resulting in a wider distribution, larger populations, and more stable communities. In contrast, specialist species have a narrow ecological niche, relying on specific resources and possessing weaker adaptability, which leads to a more limited distribution, smaller populations, and greater sensitivity to environmental changes (Pandit et al. 2009; Wilson and Hayek 2015). Investigating how different microbial taxa respond to environmental disturbances is crucial for deepening our understanding of the intrinsic relationship between biodiversity and ecosystem stability (Xue et al. 2018).

Ensuring the freshness of fecal samples is a critical step for accurately characterizing gut microbiota in birds. To obtain an accurate and unbiased assessment of this question, we conducted an experiment at Nanchang Zoo in Jiangxi Province. In the zoo, we were able to obtain the freshest fecal samples, and assess the impact of different storage times on the gut microbiota of captive migratory birds using absolute and relative quantification methods. The aims of our study were to 1) assess the effect of host species on gut microbiota; 2) examine the impact of storage time on fecal microbial community, including potential interactions between species identity and storage time; and 3) identify the classification of bacteria significantly altered by storage time (e.g., high-abundance vs. low-abundance taxa, generalists vs. specialists).

## Materials and methods

### Fecal sample collection

Fecal samples were collected from 9 captive migratory bird species at Nanchang Zoo (28°37′10″N, 115°52′19″E), Jiangxi Province, between May and June 2024, including 3 Red-crowned Cranes (*n* = 5, *Grus japonensis*, GJ), 2 Black-necked Cranes (*n* = 7, *Grus nigricollis*, GN), two Grey-crowned Cranes (*n* = 7, *Balearica regulorum*, BR), 3 Siberian Cranes (*n* = 8, *Leucogeranus leucogeranus*, LL), one White-naped Crane (*n* = 6, *Grus vipio*, GV), 7 Black Swans (*n* = 7, *Cygnus atratus*, CA), 4 Swan Geese (*n* = 4, *Anser cygnoides*, AC), one Tundra Swan (*n* = 1, *Cygnus columbianus*, CC), and one Bar-headed Goose (*n* = 1, *Anser indicus*, AI). The cranes were kept separately in different cages, while the geese shared a habitat near a pool. We observed defecation events outside the cage or by the pool to ensure the prompt collection of fresh fecal samples. Fecal samples were collected using tweezers or sterile swabs, avoiding any portions that had contacted the ground. Each of the 46 samples was divided into 4 portions. One portion was immediately frozen in liquid nitrogen (0 h) and designated as the fresh control, representing the baseline composition of the sample. The remaining 3 portions were stored at a temperature of 16 °C for 1, 2, and 4 h, respectively, before subsequently being transferred to liquid nitrogen. To control for experimental temperature, the thermostat was consistently set at 16 °C throughout the experiment, which was based on the average maximum daytime temperature recorded during the previous field collections of migratory bird feces. The entire process from defecation to initial storage—either in liquid nitrogen or temporarily at 16 °C—was completed within 5 min. In total, 184 samples were collected. After being transported to the laboratory, the fecal samples were stored at −80 °C before further analysis.

### DNA extraction and illumina-based 16S rRNA gene sequencing

Absolute quantification of bacterial 16S rRNA amplicons was performed by Majorbio Bio-Pharm Technology Co., Ltd. (Shanghai, China). Depending on the available amount, we used 50 to 190 milligrams of feces per sample for DNA extraction, some using a cotton swab. DNA was extracted using the FastPure Feces DNA Isolation Kit (Shanghai Major Yuhua), and its integrity was assessed by gel electrophoresis on a 1% agarose gel. The DNA concentration was determined with a NanoDrop2000 spectrophotometer (Thermo Scientific Inc., USA). Twelve distinct spike-in sequences at 4 different concentrations (10^3^, 10^4^, 10^5^, and 10^6^ copies of internal standards) were added to the sampled DNA pools. Each spike-in sequence contained conserved regions matching those of selected natural 16S rRNA genes. Additionally, artificial variable regions were incorporated to serve as internal standards for absolute quantification. The V3-V4 region of the bacterial 16S rRNA gene was amplified using forward 338F (5′-ACTCCTACGGGAGGCAGCAG-3′) and reverse 806R (5′-GGACTACHVGGGTWTCTAAT-3′) primers (Liu et al. 2016). Each PCR reaction contained a 20 µL PCR mix with 10 μL 2×Pro Taq, 0.8 µL of forward primer (5 μM), 0.8 µL of reverse primer (5 μM), 10 ng of template DNA, and nuclease-free water, and was conducted on an ABI GeneAmp 9700 (ABI, CA, USA). The amplification program included an initial 3-min denaturation step at 95 °C, followed by 29 cycles of 95°C for 30 s, 53 °C for 30 s, and 72 °C for 1 min, and a final elongation of 10 min at 72 °C. The amplicons were extracted from 2% agarose gels, purified with the AxyPrep DNA gel extraction kit (Axygen, USA) following the manufacturer's protocol, and their concentrations were measured using QuantiFluor™-ST (Promega, USA). The purified amplicons were pooled in equimolar amounts and paired-end sequenced on an Illumina Nextseq2000 platform according to the standard protocols.

The raw sequence data reported in this study have been deposited in the Genome Sequence Archive (GSA: CRA023318) at the National Genomics Data Center, China National Center for Bioinformation/Beijing Institute of Genomics, Chinese Academy of Sciences and are publicly accessible at https://ngdc.cncb.ac.cn/gsa.

#### Bioinformatics analysis

Raw reads were filtered for quality using Fastp (v0.19.6) (Chen et al. 2018), and FLASH (v1.2.11) was used to merge paired-end sequences (Magoč and Salzberg 2011) : 1) trimmed bases with quality scores below 20 from the 3′ ends of reads; truncated reads if 50 bp sliding window average quality <20; discarded reads <50 bp after quality control or with ambiguous bases (N). 2) merged paired-end reads with minimum 10 bp overlap; allowed max 0.2 mismatch ratio in overlap; discarded reads exceeding this. (iii) demultiplexed samples by barcodes (no mismatch) and primers (up to 2 mismatches). The DADA2 pipeline (Callahan et al. 2016) implemented in QIIME2 (https://qiime2.org) (Bolyen et al. 2019) was used to identify amplicon sequence variants (ASVs) at 100% sequence similarity. Then, ASVs assigned to spike-in sequences were filtered out, and read counts for the remaining ASVs were quantified. Standard curves (based on read counts versus spike-in DNA copy number) were generated for each sample. These were then used to determine the quantitative abundance of each ASV. The quantification was achieved by the elution volume, resulting in abundance units expressed as copies per microliter (copies/μL). Taxonomic classification of ASVs was performed using the Naive Bayes classifier implemented in QIIME2 with the SILVA 16S rRNA database (v138) (Quast et al. 2012). Finally, the absolute copy numbers of ASVs were corrected based on the ribosomal RNA operons database (*rrn*DB) to obtain a more accurate absolute abundance (Stoddard et al. 2015).

#### Microbial taxa classification

For an individual sample, the relative abundance of an Amplicon Sequence Variant (ASV) was categorized into one of 3 distinct ranges: ≤0.01%, 0.01–1%, or ≥1%. However, across different samples, the relative abundance of the same ASV could vary across these ranges (Logares et al. 2014; Liu et al. 2017; Zhang et al. 2018). To systematically analyze these variations, we classified all ASVs into 6 mutually exclusive categories based on their relative abundance, following established methodologies (Dai et al. 2016; Xue et al. 2018): 1) *always rare taxa* (ART), defined as ASVs with a relative abundance ≤0. 01% in all samples; 2) *always abundant taxa* (AAT), defined as ASVs with a relative abundance ≥1% in all samples; 3) *moderate taxa* (MT), defined as ASVs with a relative abundance consistently between 0. 01% and 1% in all samples; 4) *conditionally rare taxa* (CRT), defined as taxa with a relative abundance below 1% in all samples and ≤0.01% in some samples; 5) *conditionally abundant taxa* (CAT), defined as taxa with a relative abundance >0.01% in all samples and ≥1% in some samples but never rare (≤0.01%); and 6) *conditionally rare or abundant taxa* (CRAT), defined as taxa with a relative abundance varying from rare (≤0.01%) to abundant (≥1%). In this classification, AAT, CAT, and CRAT were classified as abundant taxa, while ART and CRT were regarded as rare taxa (Xue et al. 2018).

The niche breadth (*B*) index (Levins 1968) was calculated for the abundant and rare taxa. The formula is as follows:


$$B_{j}=\frac{1}{\sum_{i=1}^{N}\;P_{ij}^{2}}$$

where $B_{j}$ is the niche breadth and $P_{ij}\;$ is the relative abundance of ASV *j* in species *i*, and *N* represents the total number of species (*N* = 7). We calculated the *B* index based on the species abundance and evenness for each species and calculated the occurrence of ASVs generated by the simulation of 1,000 permutations (permatswap permutation algorithms) using the EcoIUtils R package (Salazar 2015). An ASV was defined as generalist species if the observed niche breadth exceeded the upper 95% confidence interval, and it was defined as specialist species if the observed niche breadth fell below the lower 95% confidence interval. The ASVs with an observed niche breadth within the 95% confidence interval were considered neutral taxa (Wu et al. 2017).

#### Statistical analysis

A total of 25,062,804 raw sequences were generated, with an average of 136,955 reads per sample. After quality filtering, 23,092,647 valid sequences remained. Subsequent denoising with DADA2 yielded 7,583,008 high-quality sequences, again averaging 41,212 reads per sample. The ASV feature sequences were further screened to remove contaminating sequences, such as mitochondria and chloroplasts. After obtaining the corrected data, low abundance ASVs with an occurrence frequency of <1% across all samples and a relative abundance below 0.01% were excluded, and finally 471 ASVs were retained for absolute abundance analysis. The relative abundance was calculated by normalizing the absolute abundance values. Fecal samples collected from the same individual at 0 h showed substantial variation in microbial composition. These initial differences allowed us to examine how fecal microbiota change over storage time. Therefore, analyses of storage time effects were based on all successfully sequenced samples (*N* = 182), excluding one lost sample and one sequencing failure. To avoid potential bias introduced by repeated sampling from the same individuals, only the first fecal sample from 21 individuals was used to compare the microbiota composition across species at 0 h.

Microbial diversity and community composition were analyzed using vegan package (Oksanen et al. 2024) in R (v4.4.1). Alpha diversity was assessed based on observed ASVs to evaluate community richness, and the Shannon index was used to quantify community diversity at the level of ASVs. Principal coordinate analysis (PCoA) based on the Bray–Curtis distance was conducted at the ASV level to assess microbial beta diversity. Permutational multivariate analysis of variance (PERMANOVA) was performed using the adonis function in the vegan package with 999 permutations to evaluate the between-group differences in microbial communities. After testing for normality (Shapiro–Wilk test) and homogeneity of variance (Bartlett test), the alpha diversity across different species, as well as the absolute and relative abundances difference among species at the phylum level, were analyzed by the non-parametric Kruskal–Wallis test combined with Dunn's multiple comparisons. Differences in alpha diversity across different storage times were evaluated using one-way ANOVA with Tukey's post hoc test. Beta diversity was compared pairwise using PERMANOVA, and all *P*-values were adjusted using the FDR method unless otherwise specified. ImageGP (https://www.bic.ac.cn/ImageGP) (Chen et al. 2022) was used to plot bar diagrams at the phylum level to display bacterial composition and their absolute and relative abundance among different species. Boxplots were also generated using ImageGP, and other figures were generated using the R package ggplot2 (Wickham 2016). To evaluate the effect of storage time on microbial abundance, we constructed a series of univariate linear regression models for each ASV, using storage time as the explanatory variable and the log10(abundance + 1) transformed abundance as the response variable. All analyses were performed in R (v4.4.1).

## Results

### Relative vs. absolute quantitation

**Figure 1:**
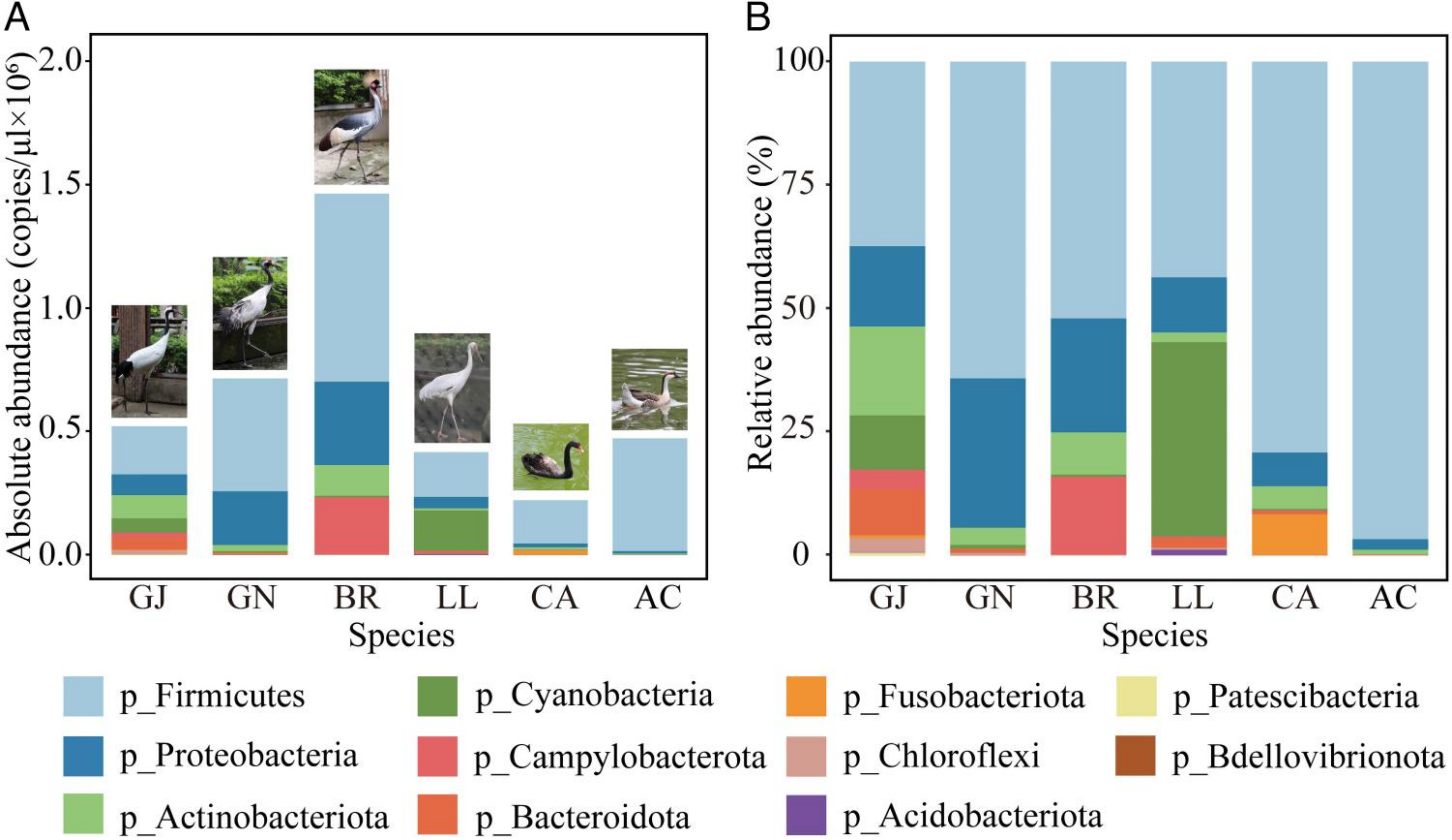
Community composition. Absolute abundance (A) and relative abundance (B) composition of bacterial communities at the phylum level among species at 0 h. Each column represents the average value of each species (GJ, *Grus japonensis*; GN, *Grus nigricollis*; BR, *Balearica regulorum*; LL, *Leucogeranus leucogeranus*; CA, *Cygnus atratus*; AC, *Anser cygnoides*).

In the 6 bird species examined, the gut microbiota was predominantly composed of bacteria from the phyla Firmicutes and Proteobacteria. The dominant microbial taxon varied across species depending on the quantification method used. For instance, based on absolute abundance, Firmicutes (763,405.73 copies/μL) and Proteobacteria (340,231.03 copies/μL) were most abundant in the BR species (Fig. 1A). In contrast, under relative abundance, Firmicutes bacteria (96.74%) were most abundant in the AC species, while Proteobacteria (30.30%) dominated in the GN species (Fig. 1B).

Given absolute abundance offers more comprehensive information than relative measures, our subsequent analyses were primarily based on absolute abundance. A total of 11 microbial phyla were detected across all 0 h samples. Significant interspecies variations in the absolute abundance were observed among species, with the BR species (1,468,730.82 copies/μL) exhibiting the highest absolute abundance, followed by the GN species (718,518.10 copies/μL), whereas the CA species (222, 248.95 copies/μL) and AC species (473,116.40 copies/μL) displayed the lowest absolute abundance. The 5 most abundant phyla—Firmicutes, Proteobacteria, Actinobacteriota, Cyanobacteria, and Campylobacterota—together accounted for 96.13% of the total absolute copy number, which the remaining 6 phyla constituted only 3.87%. As for relative abundance, the microbial community composition varied across species, although Firmicutes consistently being the most relatively abundant phylum in all species.

### Effect of species identity on microbiota diversity

**Figure 2:**
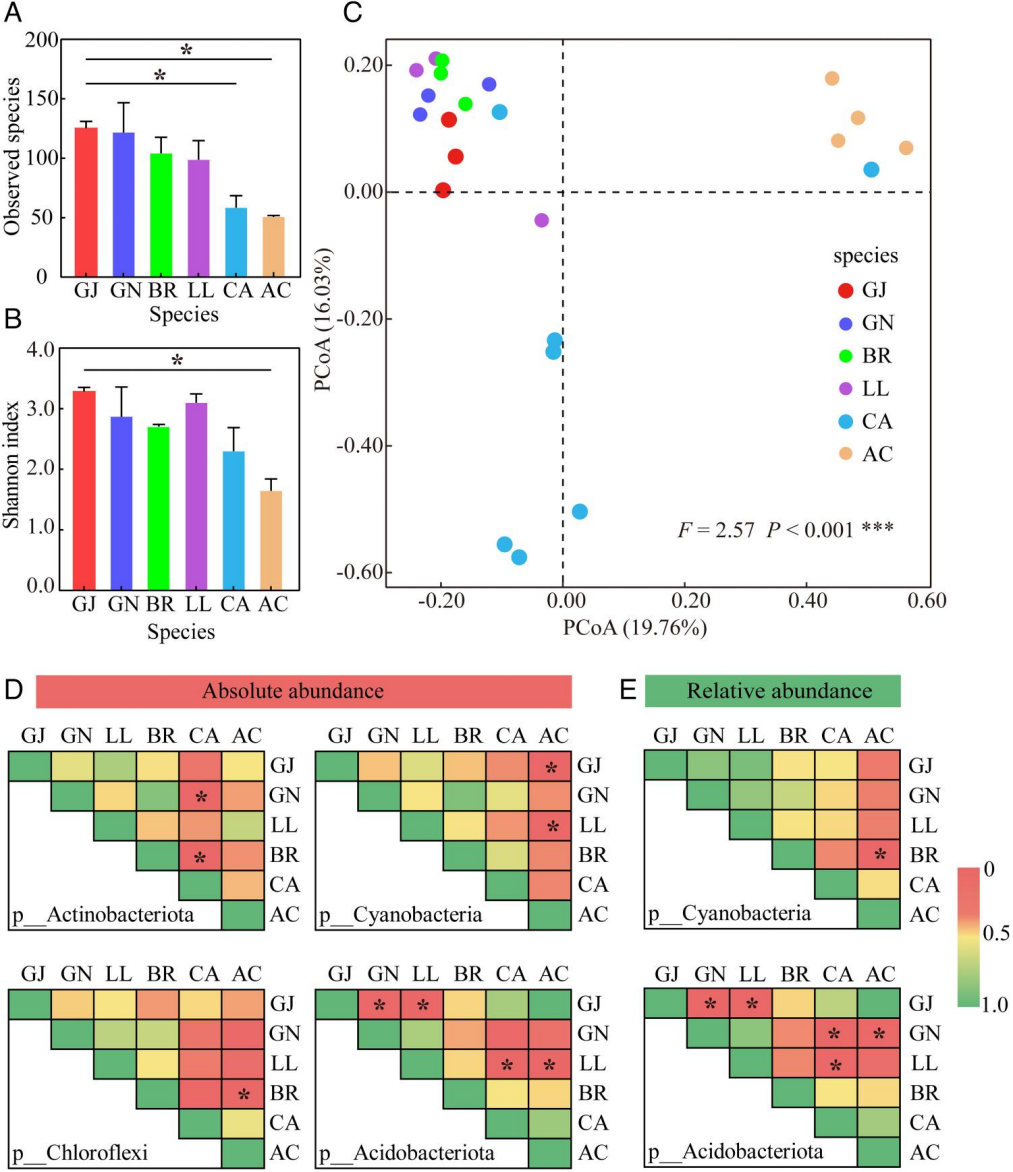
Alpha diversity, beta diversity, and phylum-level microbial community composition among species at 0 h were compared. Observed species (A) and Shannon index (B) revealed the richness and diversity of the microbial community at ASV level. PCoA plot based on the Bray-Curtis dissimilarity matrix showed the differences in microbial community composition (C). Absolute abundance of the 4 phyla exhibited significant differences among species (D), while relative abundance showed 2 phyla differed significantly among species (E). (GJ, *Grus japonensis*; GN, *Grus nigricollis*; BR, *Balearica regulorum*; LL, *Leucogeranus leucogeranus*; CA, *Cygnus atratus*; AC, *Anser cygnoides*). The asterisks represent statistical significance, *P < 0.05, ***P < 0.001.

Significant differences were observed in alpha diversity (Kruskal–Wallis test observed ASVs: *P* = 0.023; Shannon: *P* = 0.027) of gut microbiota among different species (Fig. 2A,B). Specifically, the GJ species exhibited significantly higher observed ASVs and Shannon index values compared to the CA and AC species (ASVs, GJ vs. CA *P* = 0.046, GJ vs. AC *P* = 0.016; Shannon, GJ vs. AC *P* = 0.023). In the beta diversity analysis, principal coordinates analysis (PCoA) based on Bray-Curtis distances revealed significant interspecies differences in microbial community composition (PERMANOVA, *F* = 2.572, *P* < 0.001, Fig. 2C). Samples from the 4 crane species clustered closely together, indicating similar microbial community composition, whereas samples from CA and AC species were more dispersed and exhibited significantly different microbial community compositions (pairwise PERMANOVA, *F* = 3.75, *P* = 0.016). Notably, the microbial community structure of the CA species differed significantly from that of the GJ, GN, BR, and LL species (pairwise PERMANOVA, CA vs. GJ *F* = 2.32, *P* = 0.022; CA vs. GN *F* = 1.95, *P* = 0.021; CA vs. BR *F* = 2.35, *P* = 0.019; CA vs. LL *F* = 1.8, *P* = 0.047). Similarly, the AC species showed significant differences in community structure compared to each of these 4 crane species (AC vs. GJ *F* = 7.04, *P* = 0.026; AC vs. GN *F* = 5.08, *P* = 0.029; AC vs. BR *F* = 6.03, *P* = 0.036; AC vs. LL *F* = 5.31, *P* = 0.034) (Supplementary Table S1).

A total of 203ASVs were identified in the GN species, 192 in the GJ species, 188 in the LL species, 171 in the BR species, 159 in the CA species, and 95 in the AC species (Supplementary Figure S1). Of the total 417 ASVs identified, only 13 ASVs (3.12%) were shared across all 6 species. Nineteen ASVs were shared between the CA and AC species, while twenty ASVs were shared among the crane species. Each species harbored a number of unique ASVs, with 38 (9.11%) in GJ, 28 (6.71%) in BR, 27 (6.47%) in GN, 17 (4.08%) in CA, 15 (3.60%) in LL, and 14 (3.36%) in AC.

Of the 4 phyla identified, 4—Actinobacteriota, Cyanobacteria, Chloroflexi, and Acidobacteriota—showed significant differences in absolute abundance among species, while only Chloroflexi and Acidobacteriota showed significant variation in relative abundance. Specifically, the absolute abundance of Actinobacteriota in GN and BR was significantly higher than those in CA. Cyanobacteria was more abundant in GJ and LL than in AC, which Acidobacteriota was significantly higher in LL than those in CA, AC, and GJ. For Cyanobacteria and Acidobacteriota, the relative abundance of Cyanobacteria was significantly higher in BR than in AC, while Acidobacteriota was significantly higher in LL than in GJ and AC and significantly higher in GN than in GJ, CA, and AC (Fig. 2D,E).

### Effect of storage time on gut microbiota diversity

**Figure 3:**
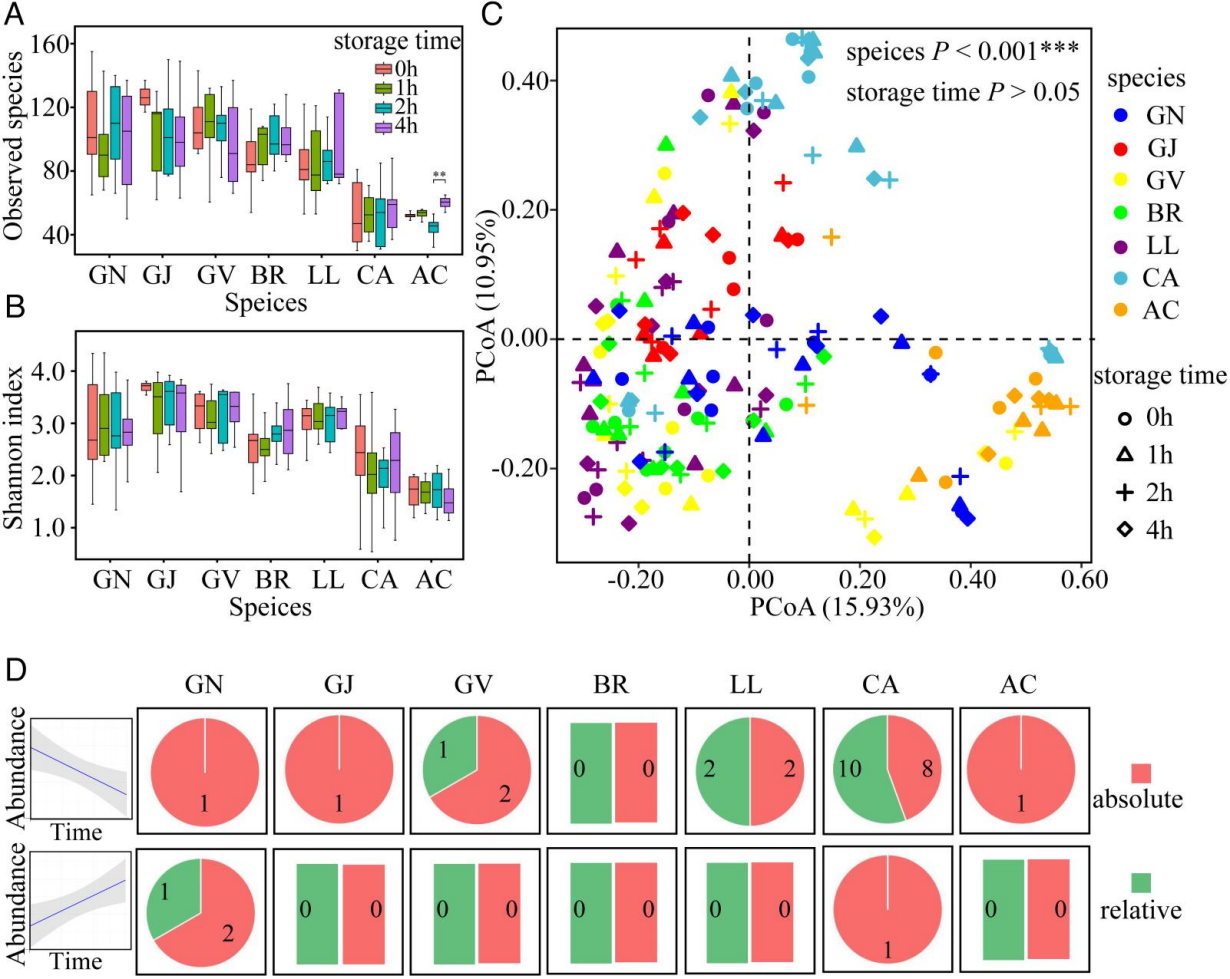
Gut microbiota among species at different storage times (0, 1, 2, and 4 h). Observed ASVs (A) and Shannon index (B) showed the richness and diversity of microbial communities at ASV level. PCoA plot based on the Bray-Curtis dissimilarity matrix showed the microbial composition (C). Linear regression analyses showed the relationship between absolute and relative abundance and storage time at ASV level (D) (The number indicated the amount of change in ASVs). (GJ, *Grus japonensis*; GN, *Grus nigricollis*; BR, *Balearica regulorum*; LL, *Leucogeranus leucogeranus*; GV, *Grus vipio*; CA, *Cygnus atratus*; AC, *Anser cygnoides*; CC, *Cygnus columbianus*; AI, *Anser indicus*). The asterisks represent statistical significance, ** *P* < 0.01, ***P < 0.001.

Except for AC species, no significant differences were observed in both the number of observed ASVs and Shannon index among species at different storage times (ANOVA, all *P* > 0.05; Fig. 3A,B). In the AC species, the observed species number at 2 h was significantly lower than that at 4 h (ANOVA, *P* = 0.007). PCoA revealed that the microbial community composition remained similar across storage times within each species, and no significant differences were detected (PERMANOVA *F =* 0.28, *P* > 0.05; Fig. 3C). However, significant differences in microbial community structure were observed among species and their interaction with storage time (PERMANOVA species *F =* 9.28, *P* < 0.001; species × storage time *F =* 2.15, *P* < 0.001). As only a single sample was available for the Tundra Swan, and Bar-headed Goose, these were analyzed separately. The results showed a consistent pattern—microbial communities did not vary across storage times but did differ among species (Supplementary Figure S2).

Linear regression analysis was conducted to examine the relationship between storage time and both the absolute and relative abundance of ASVs (Fig. 3D). At the ASV level, analysis of absolute abundance showed that across all 7 species, 15 ASVs significantly decreased with storage time, while 3 ASVs significantly increased. In contrast, the analysis of relative abundance revealed that 13 ASVs significantly decreased with storage time, while only 1 ASVs significantly increased. Furthermore, the number of ASVs that significantly changed with storage time varied among species. In the AC species showed the absolute abundance of 9 ASVs changed significantly with storage time, but no ASVs changed significantly in BR species.

### Classification of microbial taxa across different species

**Figure 4:**
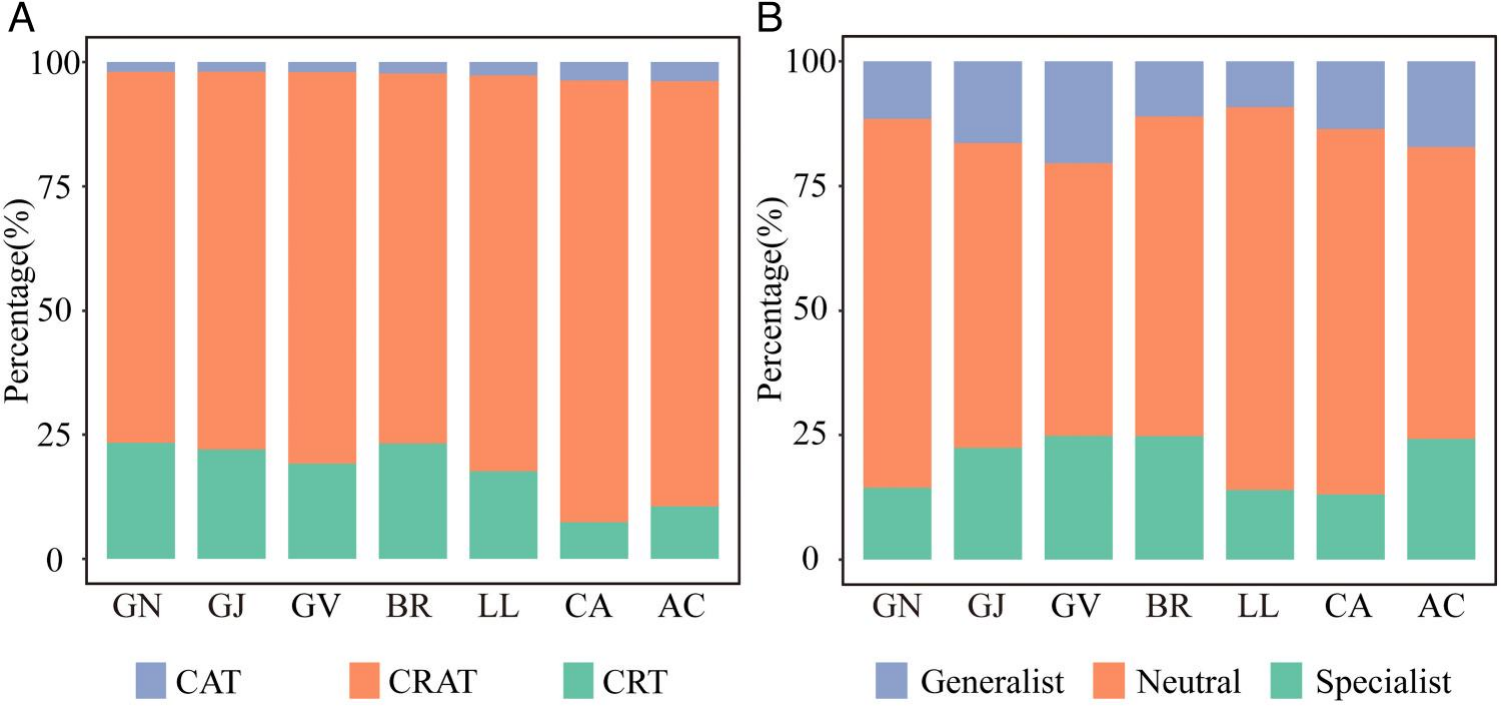
Percentage composition of different classifications across species based on ASV levels. Percentage of abundant and rare taxa (A), generalists and specialists among different species (B). Each bar graph represented the average percentage composition of all samples for each species. (GN, *Grus nigricollis*; GJ, *Grus japonensis*; GV, *Grus vipio*; BR, *Balearica regulorum*; LL, *Leucogeranus leucogeranus*; CA, *Cygnus atratus*; AC, *Anser cygnoides*).

According to the classification method described above, all bacterial ASVs across the 7 species were assigned to 3 taxa categories —CRT, CRAT, and CAT (Fig. 4A) —and grouped into 3 categories based on the niche breadth index: generalist, specialist and neutral (Fig. 4B). Specifically, the CAT group was the least abundant across all species, with only 2 ASVs detected per species. CRAT comprised more than half of the ASVs in the GN (74.72%) species, GJ (76.09%) species, GV (78.83%) species, BR (74.51%) species, LL (79.72%) species, CA (88.96%) species and AC (85.63%) species. The proportions of generalists and specialists were relatively low in each species. The proportion of generalist ASVs were 11.53% in GN, 16.38% in GJ, 20.44% in GV, 11.07% in GN, 9.18% in LL, 13.57% in CA, and 19.17% in AC. Correspondingly, the proportion of specialist ASVs were 14.41% in GN, 22.41% in GJ, 24.82% in GV, 24.76% in GN, 13.95% in LL, 13.07% in CA, and 24.22% in AC.

Linear regression analysis showed that the absolute abundance of 18 ASVs and the relative abundance of 14 ASVs changed significantly (increased or decreased) with storage time, with a total of 21 ASVs showed significant changes (Fig. 3D). CAT and CRAT were classified as abundant taxa, whereas CRT was classified as rare taxa. Among the 21 ASVs with altered abundance, 13 ASVs (62%) were classified as conditionally rare taxa (CRT) and 8 ASVs (38%) were conditionally abundant taxa (CRAT). In addition, 6 ASVs (29%) were specialist species, and 2 ASVs (10%) was generalist species (Supplementary Tables S2 and S3).

## Discussion

The gut microbiota, often referred to as the second genome, can assist the host in adapting to various environmental changes by regulating metabolic functions (Lu et al. 2024; Park and Do 2024; Zhang et al. 2024). Verifying the freshness and accuracy of fecal samples is therefore crucial for reliably assessing microbial community composition and understanding host–microbe interactions in ecological and health-related studies. We analyzed fecal samples stored at 16 °C for 1, 2 and 4 h from 9 captive migratory bird species in the zoo using both absolute and relative quantification methods to assess the impact of storage time on gut microbiota stability. Apart from AC species, which showed a significant difference in alpha diversity (Observed species) between 2 and 4 h, other species exhibited no significant effect of storage time on fecal microbiota. Although most fecal microbiota remained stable shortly after defecation, relatively few ASVs were significantly affected by storage time. Additionally, substantial discrepancies were observed between results obtained from absolute versus relative quantification. These findings provide valuable guidance for field collection of avian fecal samples.

Previous studies have also shown significant differences between microbial community profiles derived from absolute and relative abundance (Vandeputte et al. 2017; Tkacz et al. 2018). In this study, analyses based on absolute and relative abundance yielded significantly inconsistent conclusions regarding dominant phyla, species differences, and storage time effects. This result highlights the limitations of relative abundance analysis alone, as its ratio-base nature may underestimate actual microbial community differences. In contrast, absolute abundance is more sensitive to true biological changes and can provide additional insights into microbial load and dynamics. Therefore, combining absolute and relative abundance methods can help to more accurately and reliably analyze the dynamics of the intestinal microbiome.

Numerous factors influence gut microbiota, with host diet and phylogeny identified as the main drivers of microbial diversity (Youngblut et al. 2019). Host diet primarily accounts for variation in alpha diversity, while both diet and phylogeny contribute to beta diversity differences. In this study, cranes are omnivorous, whereas black swans and swan geese are herbivorous. We found significant differences in gut microbial community structure between the herbivorous waterfowl and omnivorous cranes, which may reflect dietary differences leading to different microbial communities. In mammals, gut microbial diversity generally increases from carnivores to omnivores to herbivores (Ley et al. 2008). However, studies in birds have reported higher microbial diversity in omnivorous species compared to both herbivores and carnivores (Cho and Lee 2020; Wang et al. 2022). Consistent with these findings, our results showed greater microbial diversity in cranes than in waterfowl. This pattern may be related to the broader range of dietary resources utilized by omnivorous birds, which could promote the establishment of more functionally diverse microbial communities. Additionally, differences in gut morphology and digestive physiology between omnivores and herbivores might further contribute to the observed variation in microbial diversity.

In this study, the storage time was set to a maximum threshold of 4 h because the entire process from finding the migratory bird population to freezing the sample in liquid nitrogen did not exceed 4 h, based on the previous field collection of migratory bird feces. All species showed no significant effect of storage time on the stability of the gut microbiota, except for AC species at 2 and 4 h. It is possible that the relatively short storage duration of 4 h may not have been sufficient to induce significant changes in gut microbial communities; longer storage periods (e.g., 8 h, 24 h, or more) or higher temperatures might be necessary to observe variations. For example, significant shifts in fecal microbial communities were detected in springbok after 3–4 days and in giraffe after 1 week of environmental exposure (Menke et al. 2015). Likewise, microbiome differences emerged in human and dog feces after 1 week at room temperature (Song et al. 2016).

We found that relatively few ASVs were significantly affected by storage time, suggesting that while most fecal microbiota remain relatively stable shortly after defecation, a small subset of more sensitive taxa exhibit changes. Most ASVs that responded to storage time were classified as conditionally rare taxa (CRT), indicating that low-abundance ASVs are more susceptible to fluctuations during environmental exposure, while dominant members of the community tend to remain stable. This finding aligns with previous studies suggesting that rare species were more sensitive to environmental fluctuations than abundant taxa (Xue et al. 2018). One possible explanation is that many rare species tend to grow more slowly and have specialized metabolic requirements, making them more susceptible to changes in environmental conditions, such as oxygen exposure or nutrient availability during storage (Logares 2015). Low-abundance and rare bacteria are often more sensitive to environmental changes, and their abundance may change significantly if samples are not preserved in time.

## Supplementary Material

zoaf066_Supplementary_Data
