# Supplementary material for: Disentangling host identity and storage time effects on gut microbiota composition in captive migratory birds using absolute and relative quantification
Source: Curr Zool. 2025 Oct 3;72(3):441–50. doi: 10.1093/cz/zoaf066 (PMC13290400; doi:10.1093/cz/zoaf066)
Supplement: zoaf066_Supplementary_Data [file zoaf066_supplementary_data.docx]

**Table S1** PERMANOVA of gut microbiome based on Bray-Curtis dissimilarity among different species (GJ, *Grus japonensis*; GN, *Grus nigricollis*; BR, *Balearica regulorum*; LL, *Leucogeranus leucogeranus*; CA, *Cygnus atratus*; AC, *Anser cygnoides*). *P* values were corrected for FRD.

| Group | R^2^ | F | P.adjust | Sig |
| --- | --- | --- | --- | --- |
| GJ vs GN | 0.257 | 1.381 | 0.2 | ns |
| GJ vs BR | 0.339 | 2.054 | 0.1 | ns |
| GJ vs LL | 0.312 | 1.815 | 0.1 | ns |
| GJ vs CA | 0.225 | 2.326 | 0.022 | * |
| GJ vs AC | 0.585 | 7.04 | 0.026 | * |
| GN vs BR | 0.228 | 1.184 | 0.4 | ns |
| GN vs LL | 0.22 | 1.127 | 0.4 | ns |
| GN vs CA | 0.196 | 1.953 | 0.021 | * |
| GN vs AC | 0.504 | 5.08 | 0.029 | * |
| BR vs LL | 0.265 | 1.441 | 0.3 | ns |
| BR vs CA | 0.227 | 2.35 | 0.019 | * |
| BR vs AC | 0.547 | 6.028 | 0.036 | * |
| LL vs CA | 0.184 | 1.805 | 0.047 | * |
| LL vs AC | 0.515 | 5.311 | 0.034 | * |
| CA vs AC | 0.294 | 3.574 | 0.016 | * |

**Table S2** Linear regression analyses based on absolute abundance of ASVs and the classification of ASVs (GJ, *Grus japonensis*; GN, *Grus nigricollis*; GV, *Grus vipio*; BR, *Balearica regulorum*; LL, *Leucogeranus leucogeranus*; CA, *Cygnus atratus*; AC, *Anser cygnoides*).

| **species** | **ASV** | **Estimate** | **p.adjust** | **R_squared** | **Variation** | **Tax** | **Sign** |
| --- | --- | --- | --- | --- | --- | --- | --- |
| LL | ASV319 | -0.549 | 0.039 | 0.200 | decrease | CRT | Others |
| LL | ASV122 | -0.588 | 0.043 | 0.194 | decrease | CRAT | Others |
| GJ | ASV221 | -1.941 | 0.020 | 0.372 | decrease | CRAT | Specialist |
| GV | ASV183 | -2.317 | 0.013 | 0.271 | decrease | CRAT | Specialist |
| GV | ASV455 | -1.458 | 0.047 | 0.268 | decrease | CRT | Others |
| GN | ASV67 | 2.134 | 0.018 | 0.211 | increase | CRT | Others |
| GN | ASV249 | -1.955 | 0.019 | 0.225 | decrease | CRAT | Generalist |
| GN | ASV91 | 1.651 | 0.030 | 0.200 | increase | CRT | Generalist |
| CA | ASV55 | -0.930 | 0.001 | 0.483 | decrease | CRT | Others |
| CA | ASV459 | -1.257 | 0.026 | 0.238 | decrease | CRAT | Others |
| CA | ASV235 | -1.123 | 0.032 | 0.267 | decrease | CRAT | Specialist |
| CA | ASV23 | 0.623 | 0.039 | 0.230 | increase | CRAT | Specialist |
| CA | ASV45 | -0.459 | 0.039 | 0.229 | decrease | CRT | Others |
| CA | ASV309 | -0.749 | 0.040 | 0.227 | decrease | CRAT | Specialist |
| CA | ASV345 | -1.010 | 0.041 | 0.187 | decrease | CRAT | Others |
| CA | ASV425 | -0.681 | 0.042 | 0.223 | decrease | CRAT | Others |
| CA | ASV421 | -1.134 | 0.050 | 0.155 | decrease | CRAT | Others |
| AC | ASV311 | -0.831 | 0.032 | 0.424 | decrease | CRT | Others |

**Table S3** Linear regression analyses based on relative abundance of ASVs and the classification of ASVs (GJ, *Grus japonensis*; GN, *Grus nigricollis*; GV, *Grus vipio*; BR, *Balearica regulorum*; LL, *Leucogeranus leucogeranus*; CA, *Cygnus atratus*; AC, *Anser cygnoides*).

| **species** | **ASV** | **Estimate** | **p.adjust** | **R_squared** | **Variation** | **Tax** | **Sign** |
| --- | --- | --- | --- | --- | --- | --- | --- |
| LL | ASV319 | -0.0001 | 0.024 | 0.235 | decrease | CRT | Others |
| LL | ASV122 | 0.0000 | 0.035 | 0.210 | increase | CRAT | Others |
| GV | ASV455 | -0.0003 | 0.049 | 0.254 | decrease | CRT | Others |
| GN | ASV67 | 0.0031 | 0.025 | 0.198 | increase | CRT | Others |
| CA | ASV55 | -0.0001 | 0.008 | 0.347 | decrease | CRT | Others |
| CA | ASV306 | -0.0006 | 0.016 | 0.285 | decrease | CRAT | Specialist |
| CA | ASV345 | -0.0004 | 0.020 | 0.251 | decrease | CRAT | Others |
| CA | ASV235 | -0.0007 | 0.028 | 0.234 | decrease | CRAT | Specialist |
| CA | ASV421 | -0.0008 | 0.033 | 0.217 | decrease | CRAT | Others |
| CA | ASV459 | -0.0015 | 0.034 | 0.232 | decrease | CRAT | Others |
| CA | ASV425 | -0.0003 | 0.038 | 0.231 | decrease | CRAT | Others |
| CA | ASV45 | -0.0002 | 0.042 | 0.225 | decrease | CRT | Others |
| CA | ASV336 | -0.0002 | 0.046 | 0.202 | decrease | CRAT | Others |
| CA | ASV79 | -0.0014 | 0.048 | 0.217 | decrease | CRT | Others |

**
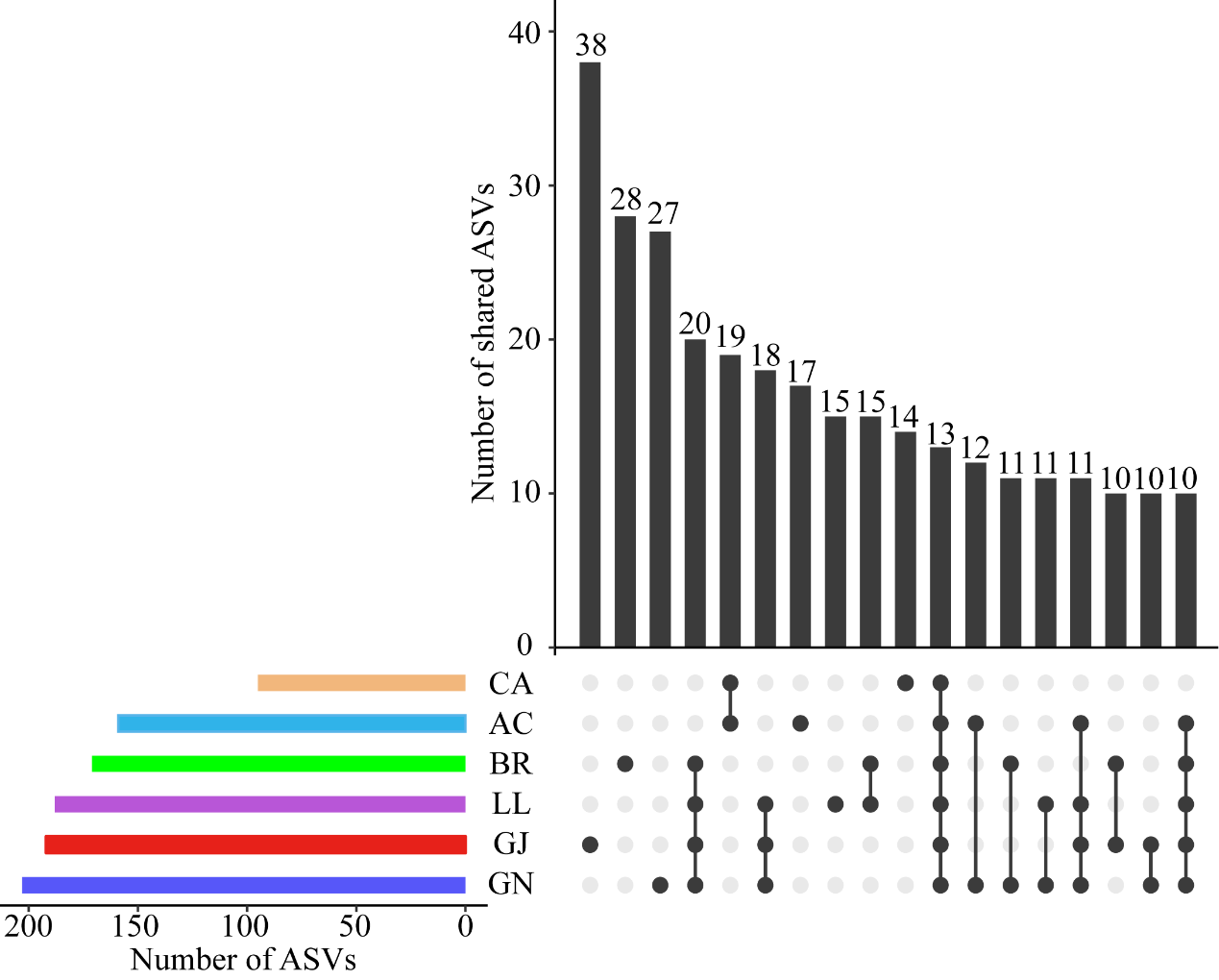
**

**FIGURE S1** The number of ASVs that were unique and shared among gut microbiome of six species (GJ, *Grus japonensis*; GN, *Grus nigricollis*; BR, *Balearica regulorum*; LL, *Leucogeranus leucogeranus*; CA, *Cygnus atratus*; AC, *Anser cygnoides*).

**
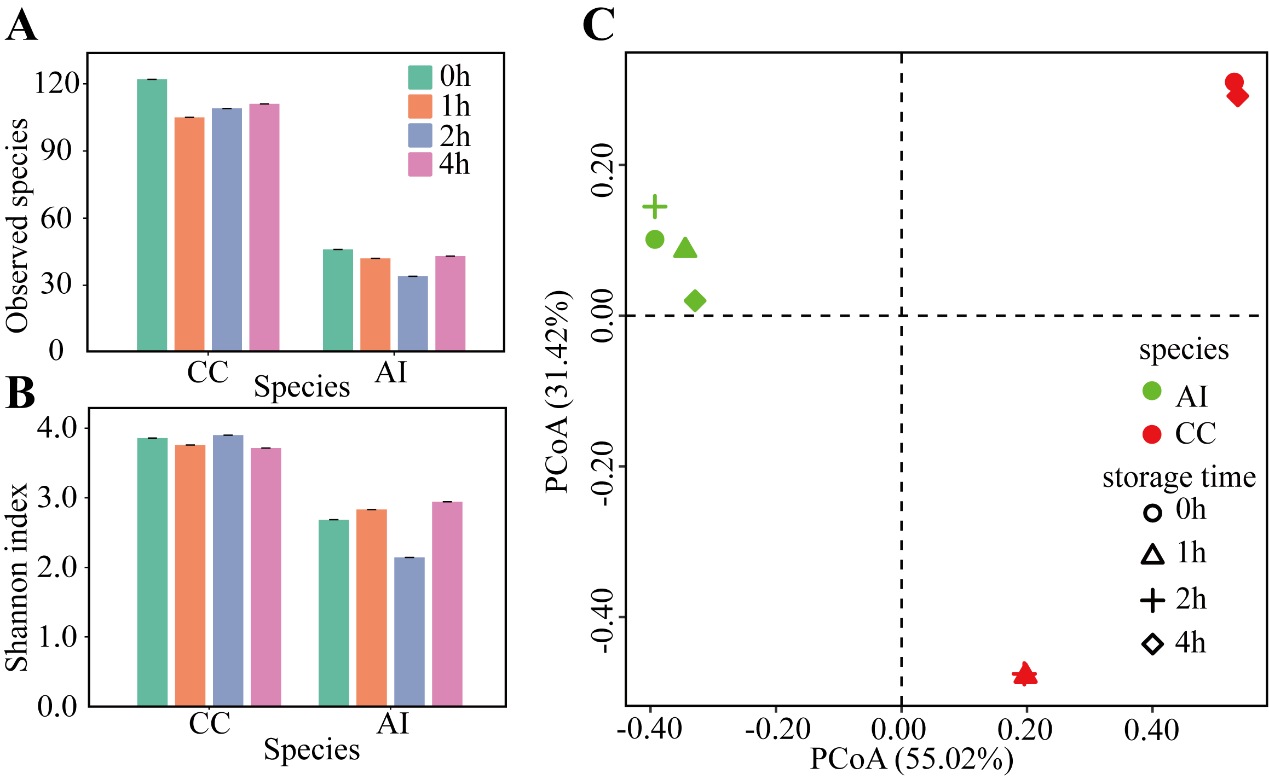
**

**FIGURE S2** Microbial community diversity and composition across different species at different storage times (0 h, 1 h, 2 h, and 4 h). Observed ASV (A) and Shannon index (B) showed the richness and diversity of microbial communities across different species at different storage times at ASV level. PCoA plot based on the Bray-Curtis dissimilarity matrix showed the microbial composition across different species at different storage times at ASV level (C). (CC, *Cygnus columbianus*; AI, *Anser indicus*).
